# Technological advances in inflammatory bowel disease endoscopy and histology

**DOI:** 10.3389/fmed.2022.1058875

**Published:** 2022-11-11

**Authors:** Ludovico Alfarone, Tommaso Lorenzo Parigi, Roberto Gabbiadini, Arianna Dal Buono, Antonino Spinelli, Cesare Hassan, Marietta Iacucci, Alessandro Repici, Alessandro Armuzzi

**Affiliations:** ^1^Department of Biomedical Sciences, Humanitas University, Milan, Italy; ^2^IBD Center, IRCCS Humanitas Research Hospital, Milan, Italy; ^3^Colon and Rectal Surgery Division, IRCCS Humanitas Research Hospital, Milan, Italy; ^4^Endoscopy Unit, IRCCS Humanitas Research Hospital, Milan, Italy; ^5^Institute of Immunology and Immunotherapy, University of Birmingham, Birmingham, United Kingdom; ^6^Department of Gastroenterology, University Hospitals Birmingham NHS Foundation Trust, Birmingham, United Kingdom

**Keywords:** artificial intelligence, endoscopy, inflammatory bowel disease, histology, computeraided diagnosis system

## Abstract

Accurate disease characterization is the pillar of modern treatment of inflammatory bowel disease (IBD) and endoscopy is the mainstay of disease assessment and colorectal cancer surveillance. Recent technological progress has enhanced and expanded the use of endoscopy in IBD. In particular, numerous artificial intelligence (AI)-powered systems have shown to support human endoscopists' evaluations, improving accuracy and consistency while saving time. Moreover, advanced optical technologies such as endocytoscopy (EC), allowing high magnification *in vivo*, can bridge endoscopy with histology. Furthermore, molecular imaging, through probe based confocal laser endomicroscopy allows the real-time detection of specific biomarkers on gastrointestinal surface, and could be used to predict therapeutic response, paving the way to precision medicine. In parallel, as the applications of AI spread, computers are positioned to resolve some of the limitations of human histopathology evaluation, such as interobserver variability and inconsistencies in assessment. The aim of this review is to summarize the most promising advances in endoscopic and histologic assessment of IBD.

## Introduction

Inflammatory bowel diseases (IBD), encompassing Crohn's disease (CD) and ulcerative colitis (UC), are chronic inflammatory gastrointestinal disorders with significant impact on quality of life and general well-being, and with a potential to develop disabling complications (1, 2). Symptom-based therapeutic strategies have been shown to fail in altering the natural course of IBD, while the treat-to-target approach has shown to improve long-term outcomes (3). This requires setting predefined objective targets, such as biomarker levels and endoscopic remission (ER), to be achieved by therapy (4). In the last few years, this approach has evolved further aiming for increasingly strict targets, including histological remission (HR) as an adjunctive desirable goal beyond ER (5). Thus, tight and objective assessment of intestinal inflammation is crucial to optimize treatment appropriately.

Endoscopy represents the gold standard for disease activity assessment and colorectal cancer surveillance (6). However, a reliable evaluation of inflammation as well as the detection of precancerous lesions require time and expert hands (7). In daily practice endoscopy and histology reports are often descriptive, with little use of validated scores and thus limiting comparison. Nevertheless, even when scores are applied by experts, interobserver variability remains high, and thus reproducibility is poor (8–10).

Recent applications of artificial intelligence (AI) in image analysis promise to overcome some of the limitations of human endoscopists and pathologists, chiefly speeding up and standardizing evaluations; but also enhancing conventional assessment. Other remarkable technological advances, such as endocytoscopy (EC) and molecular imaging with use of probe-based confocal laser endomicroscopy (pCLE), promise to expand the traditional role of endoscopy in IBD, bridging it to histology. This review aims to summarize the latest technological progress in IBD endoscopy and histology, and future trends in the field.

## AI for endoscopy

Clinical management according to the treat-to-target strategy requires a reliable endoscopic evaluation (5). However, this requires dedicated training and even in expert hands remains burdened by high interobserver variability and low reproducibility (8–10).

In real-world clinical practice, lack of homogeneity of assessments between endoscopists can lead to inaccurate therapeutic decisions. More so, in the setting of clinical trials, where strict evaluation and comparison are essential, central reading is often required. This process is cumbersome, expensive, and does not eliminate operator-subjectivity. Reliable assessment demands accurate procedures and detailed reports, which are time-consuming and require expertise not always available (7).

### White light endoscopy

Recently, several machine learning (ML) algorithms have been developed for assessing UC disease activity in frames or videos of colonoscopies (Table 1). Bossuyt et al. in a pilot study of 29 UC patients and 6 healthy controls presented a computer model to estimate disease activity in UC. The algorithm was based on the pixel color, the red channel of the red-green-blue (RGB) pixel values, and vascular pattern recognition in endoscopic images from colonoscopy videos performed with Pentax scopes. This computer tool provided an objective operator-independent score (Red Density^®^), which showed a significant correlation with both endoscopic (Mayo Endoscopic Subscore (MES) *r* = 0.76 and Ulcerative Colitis Endoscopic Index of Severity (UCEIS) *r* = 0.74, *p* < 0.0001) and histological scores (Robarts Histopathology Index (RHI), *r* = 0.74, *p* < 0.0001) (11). Despite some limitations, such as small sample size, single-center, use of frames rather than full videos, the AI performed well and fueled interested in the field. A larger multicenter study (PRognOstiC valuE of rEd Density in Ulcerative Colitis: PROCEED-UC; NCT04408703) is ongoing to validate this operator-independent Red Density^®^ score and assess its association with long-term outcomes. Another algorithm fitted on a prototype endoscope with a single short wave-length monochromatic LED light (Fujifilm, Tokyo, Japan) was tested by the same group (17). This technique provides a magnification up to 200 μm enabling *in vivo* real-time assessment of mucosal architecture and microscopic vascular changes, which are correlated with UC histological activity. The algorithm is based on the automated evaluation of the number of pixels with bleeding and the density of mucosal vessels per pixel. In a pilot study using endoscopic images and bioptic samples from 58 patients, this automated AI-powered tool predicted HR (according to the Geboes score) with higher accuracy (86%) than MES (74%) and UCEIS (79%) (17). Although further larger validation trials are necessary, these kind of algorithms may allow an objective and accurate real-time assessment of histological activity without any additional need for a contrast agent.

**Table 1:** Studies of AI application for IBD endoscopy.

**Author (Year)**	**Study design**	**Population**	**Outcome**	**Results**
Bossuyt et al. (11)	Prospective cohort study	29 UC patients and 6 controls	To assess an algorithm (RD) based on red–green-blue pixel values, and vascular pattern recognition from endoscopic images to predict endoscopic and histological activity	The RD algorithm agreed well with MES (*r* = 0.76, *p* < 0.0001), UCEIS (*r* = 0.74, *p* < 0.0001) and RHI (*r* = 0.74, *p* < 0.0001)
Bossuyt et al. (11)	Prospective cohort study	Colonoscopy images and biopsies from 58 UC patients	To test an algorithm based on the number of bleeding pixels and the density of vessels per pixel from endoscopic images to predict histological activity	The automated algorithm detected HR with a better accuracy (sensitivity 79% and specificity 90%) than MES (sensitivity 98% and specificity 61%) and UCEIS (sensitivity 95% and specificity 69%)
Takenaka et al. (12)	Prospective cohort study	40,758 colonoscopy images and 6885 biopsies from 2012 UC patients	To evaluate a DNN algorithm trained on endoscopic images of UC to predict endoscopic and histological remission	The DNN system predicted ER and HR with 91 and 92.9% accuracy, respectively. It had good correlation with UCEIS (ICC 0.917)
Takenaka et al. (13)	Prospective cohort study	Full endoscopy videos and 900 biopsies from 770 UC patients	To test a DNN algorithm trained on endoscopic videos of UC to predict endoscopic and histological activity	The DNN system had a sensitivity of 97.9% and a specificity of 94.6% for predicting HR. High agreement with UCEIS (ICC 0.927)
Gottlieb et al. (14)	Phase II randomized controlled trial	795 full endoscopy videos from 249 UC patients	To assess a recurrent neural network system based endoscopy videos of UC in predicting endoscopic activity	The recurrent neural network algorithm had a strong correlation with both MES (QWK 0.844) and UCEIS (QWK 0.855)
Yao et al. (15)	Phase II randomized controlled trial	315 videos from 157 UC patients	To evaluate a CNN fully automated video analysis system for grading endoscopic activity	CAD system had high sensitivity (90%) and specificity (87%); it accurately predicted MES in 82.8% of videos (κ = 0.78)
Iacucci et al. (16)	Prospective cohort study	1090 videos from 283 UC patients	To test a CNN to assess endoscopic activity, predict histological activity and outcome from WL and VCE videos	CNN reliably detected ER in both WL (72% sensitivity, 87% specificity) and VCE videos (sensitivity 79%, specificity 95%). Additionally, it predicted HR, with sensitivity, specificity and accuracy of 67, 86, and 81% respectively, using WL, and 73, 86, and 83% respectively, in VCE videos

AI, artificial intelligence; CAD, computer-assisted diagnosis; CNN, convolution neural network; DNN, deep neural network; ER, endoscopic remission; HR, histological remission; IBD, inflammatory bowel disease; ICC, intraclass correlation coefficient; MES, Mayo endoscopic subscore; QWK, quadratic weighted kappa, RD, red density; RHI, robarts histopathology index; VCE, virtual chromoendoscopy; UC, ulcerative colitis, UCEIS, ulcerative colitis endoscopic index of severity; WL, white light.

Unlike the above reported algorithms, all of the other published computer-aided diagnosis (CAD) systems are based on Convolutional Neural Networks (CNN) for detection of pathological features, instead of optical analysis. Takenaka et al. designed and validated a deep neural network (DNN) algorithm for determining UC activity, trained on 40,758 colonoscopy images and 6,885 biopsy specimens from 2012 UC patients. AI assessment of each image was compared with the UCEIS score reported by three expert endoscopists and the Geboes score assessed by three gastrointestinal pathologists; if in doubt, the score was discussed with a more IBD experienced pathologist (12). For each frame, the DNN detected ER with 91% accuracy. Moreover, the intraclass correlation coefficient (ICC) for UCEIS between the DNN and the endoscopist was 0.917 (95% CI 0.911–0.921), meaning human and AI-assisted scoring had very good agreement (12). Of note, the algorithm also predicted HR with 92.9% accuracy. The CAD system was subsequently retrained to evaluate directly colonoscopy videos instead of still images. The large multicentre prospective study testing it on 770 UC patients and 900 biopsies found an extremely high diagnostic performance for prediction of HR (sensitivity 97.9%, specificity 94.6%) and an excellent agreement with human experts for endoscopic scoring (ICC = 0.93) (13). This represents the first AI model to provide both an accurate assessment of endoscopic activity and a reliable prediction of histological activity from videos.

Another approach, a recurrent neural network by Gottlieb et al. was developed and trained to predict MES and UCEIS scores using 795 full-length endoscopy videos collected from a multicenter phase 2 trial of 249 UC patients from 14 countries. The agreement between AI-powered endoscopic score and human central reading was excellent, with a quadratic weighted kappa (QWK) of 0.844 (95% CI, 0.787–0.901) for MES and 0.855 (95% CI, 0.80–0.91) for UCEIS (14).

Yao et al. developed a fully automated video analysis system for grading UC activity. This tool, built using a CNN, was used to assess endoscopic disease activity in 315 videos collected from a phase II randomized controlled study with 157 UC patients. The CAD system had a good performance with a sensitivity of 90% and a specificity of 87%, correctly predicting MES in 82.8% of videos (κ = 0.78) (15).

Altogether, these promising results show how ML methods could soon replace human central reading, speeding up and simplifying clinical trials, and at the same time, improve endoscopic reporting accuracy in real-world clinical practice.

### Virtual chromoendoscopy

Implementation of high-definition (HD) endoscopes equipped with virtual chromoendoscopy (VCE) has led to a better evaluation of vascular patterns and mucosal surface characteristics, allowing distinction of mild from quiescent disease, a distinction increasingly recognized as clinically relevant (6, 18).

Iacucci et al. (16) developed the first AI-based system to assess endoscopic activity, predict histological activity and clinical outcome, from videos not only in white-light (WL) but also using VCE. They used 1,090 endoscopy videos of 283 patients from the PICaSSO prospective multicenter international study (19), to develop a CNN. This algorithm detected ER in WL videos (UCEIS ≤ 1) with 72% sensitivity, 87% specificity and an area under the ROC curve (AUROC) of 0.85, but when employing VCE (ER defined as PICaSSO ≤ 3) sensitivity improved to 79%, specificity to 95% and AUROC to 0.94. Additionally, the CAD system predicted HR (defined as Robarts Histologic index ≤ 3 and no neutrophils in lamina propria) with sensitivity, specificity and accuracy of 67, 86, and 81% respectively, using WL, and 73, 86, and 83% respectively, in VCE videos (16).

Finally, another extremely interesting area of potential AI application is detection of dysplasia and cancer. IBD patients have an increased risk of developing colorectal cancer (CRC) than the general population, with an estimated prevalence of about 3.5% in both longstanding UC and colonic CD (20, 21). To mitigate this risk, IBD patients undergo regular endoscopic surveillance to detect precursor dysplastic lesions or early CRC (6). Due to its subtle appearance, the detection of IBD-associated dysplasia remains one of the greatest challenges in IBD endoscopy and an AI support would be precious.

A first step was recently made with a computer aided detection (CADe) system, previously developed for detection of colorectal polyps in patients without IBD, re-trained on IBD polypoid lesions, including also non dysplastic lesions such as pseudopolyps and serrated epithelial changes (22). The CADe system showed a high sensitivity (95%) in detecting IBD dysplastic lesions as well as pseudopolyps and serrated epithelial changes (22) although specificity was not reported. Of note, despite the small sample size, the inflammation severity did not significantly affect detection rates. Indeed, authors reported that, of the 9 lesions missed by the IBD-CADe system, two had a MES of 3, two had a score of 2, two had a score of 1 and three had a score of 0.

Similarly to CAD systems approved for detection of colorectal polyps', AI-powered tools to assist endoscopists in IBD-surveillance would be of great help. However, the low prevalence of dysplasia in IBD patients limits collection of datasets and therefore AI training. Moreover, the fine tuning of CAD systems is significantly complicated by concurrent mucosal inflammation, which can represent a confounding factor for both humans and computers.

## Endocytoscopy

Discordance between endoscopic and histological disease activity has been widely documented and mild histological activity is often found in patients with ER. This subtle persistence of activity is associated to an higher risk of flare and complications (23, 24) and thus, there is interest in closing the gap between endoscopy and histology.

EC is a novel type of ultramagnification imaging technique that enables microscopic observation at the cellular level (25). As certain features, including nuclei shape, lumen of the glands, crypt architecture, cellular infiltration and microvessels' pattern, can be assessed *in vivo* during endoscopic procedures, EC promises to predict histologic activity without need for biopsies (26).

A pilot study comparing endocytoscopic, endoscopic and histological assessment of UC, showed that EC indeed resembles histology more than endoscopy (27). In another study of UC subjects in ER (MES 0), EC reliably differentiates those in HR from those with histologically active disease (28). Similarly, Takishima et al. (29) observed that number of Goblet cells quantified with EC predicted long-term sustained clinical remission in UC patients in ER (MES 0). Moreover, a recent study by Vitali et al. (30) showed endocytoscopic assessment of microscopic disease activity in UC was more accurate than WL endoscopy and highly correlated with histological scores; furthermore, EC was shown as reliable as histology for prediction of clinical outcomes in UC patients.

Hence, EC promises to narrow the gap between endoscopy and histology and may, one day, challenge the need for biopsy specimens. To enhance and standardize the interpretation of EC's ultra-magnified imaging regardless of the expertise of the endoscopist, AI-powered tools have been developed on EC images. A recent prospective cohort study on 145 UC patients in clinical remission showed that an AI-assisted tool, trained on EC images, predicted the risk of clinical relapse without requiring biopsies (31).

EC ultra-magnification provides cellular and microvascular assessment and can help distinguishing adenomas from non-neoplastic lesions, potentially improving cost-effectiveness by reducing unnecessary polypectomies. Kudo et al. (32) showed that an AI-assisted system (EndoBRAIN^®^) developed on 69,142 endocytoscopic images, reliably differentiated neoplastic from non-neoplastic lesions. At present, despite some promising case studies (33), CAD systems, trained on EC images, for detection of dysplastic lesions in IBD patients are not yet available, but would be highly desirable.

## AI capsule endoscopy

Video capsule endoscopy (CE) allows inspection of the whole gastrointestinal tract, and has higher accuracy in diagnosing proximal small bowel CD than magnetic resonance enterography (34). Hence, CE is indicated to exclude proximal small bowel CD in patients with marked clinical suspicion and negative imaging tests (6).

The analysis of CE recordings can be complicated and requires long time. Subtle findings like small aphthae can be missed in up to 11% of cases even after several hours of video-reviewing by experienced endoscopists (35). In this setting, AI-assisted tools have been developed to expedite CE reviewing and increase detection rate (Table 2).

**Table 2:** Key studies on AI application for capsule endoscopy (CE) in IBD.

**Author (Year)**	**Study design**	**Population**	**Outcome**	**Results**
Aoki et al. (36)	Retrospective cohort study	10,440 CE images	To assess a CNN system for automated identification of ulcers and erosions in CE images of SB	The CNN evaluated 10,440 images in 233 seconds and identified ulcers and erosions with 88.2% sensitivity and 90.9% specificity
Aoki et al. (37)	Retrospective cohort study	20 entire SB CE videos	To evaluate a CNN model as the first screening on SB CE video readings, comparing endoscopist reviewing after the CNN screening with endoscopist-alone reviewing	CNN reduced reviewing time (from 12.2 min to 3.1 for experienced operators and from 20.7 to 5.2 for trainees) without affecting detection rate of erosions and ulcers (experienced operators: 87 vs. 84%; trainees: 55 vs. 47%)
Klang et al. (38)	Retrospective cohort study	17,640 CE images from 49 CD patients	To test a CNN system for the automated identification of SB ulcers in CD on CE images	The CNN algorithm discriminated normal mucosa from ulcers with high accuracy (>95%)
Barash et al. (39)	Retrospective cohort study	17,640 CE images from 49 CD patients	To assess a CNN for grading CD ulcers on CE images	The AI-assisted tool had an overall agreement with capsule readers of 67%, with an accuracy of 91% for severe ulcers
Ferreira et al. (40)	Retrospective cohort study	8,085 CE images from CD patients	To evaluate an AI algorithm for the automated detection of erosions and ulcerations in both SB and colon CE images from CD patients.	The CNN system accurately identified both ulcers (sensitivity 83%; specificity 98%) and erosions (sensitivity 91%; specificity 93%)
Xie et al. (41)	Retrospective cohort study	2,898 CE videos	CAD system trained on CE videos vs. conventional reading, in detection and classification of SB findings	The DNN-based reading reached higher detection rate of SB findings than conventional reading (95.9 vs. 76.1%) in a less time (5.4 vs. 51.4 min)

AI, artificial intelligence; CAD, computer-assisted diagnosis; CD, Crohn's disease; CE, capsule endoscopy; CNN, convolution neural network; DNN, deep neural network; SB, small bowel.

Aoki et al. developed a deep CNN system to automatically detect small bowel pathologic findings in CE images. This AI model was trained on 5,360 small bowel CE images of ulcerations and erosions, obtained using a Pillcam SB2 or SB3 WCE device (Given Imaging, Yoqneam, Israel), and then assessed using a cohort of 10,440 images, encompassing 440 with erosions and ulcers (36). This CNN system completed the evaluation of 10,440 images in < 4 min (233 s) with high accuracy for detection of ulcers and erosions (sensitivity 88.2%, specificity 90.9%), using a cut-off value of 0.481 for the probability score (36).

The same group assessed the same CNN as first screening for small bowel CE video reviewing. They compared endoscopist-alone readings with endoscopist readings after the AI-supported screening (37) and concluded that the CNN decreased reviewing time (from 12.2 min to 3.1 for experienced operators and from 20.7 to 5.2 for trainees) without reducing the overall detection rate of erosions and ulcerations (37).

A similar CNN system was built by Klang et al. for the automated detection of small bowel ulcers in CD on CE images acquired by PillCam SBIII (Medtronic Ltd, Dublin, Ireland) and reviewed with Rapid 9 (Medtronic Ltd) capsule reading software. The training dataset included 17,640 CE images from 49 CD patients (10,249 images of normal mucosa and 7,391 images with mucosal ulcers) (38). This model achieved an accuracy >95% in distinguishing normal mucosa from ulcers and significantly reduced the reviewing time (38). Using the same dataset, a CNN-based model was trained for grading CD ulcers on CE images and then compared with capsule readers. The machine achieved an overall agreement with human readers of 67%, with an higher accuracy (91%) for classifying severe ulcerations (39).

Moreover, an AI algorithm was developed to automatically identify erosions and ulcerations in both small bowel and colon CE images from CD patients (PillCam™ Crohn's Capsule) (40). 8,085 PillCam™ Crohn's Capsule images, comprising 3,255 images of normal enteric and colonic mucosa and 4,830 of ulcers and/or erosions, were used for training and validation (40). This CNN-based model accurately detected both ulcers (sensitivity 83%; specificity 98%) and erosions (sensitivity 91%; specificity 93%) (40). These encouraging findings highlight how the implementation of AI-supported systems may enhance accuracy of CE for both CD's diagnosis and monitoring.

Very recently, Xie et al. (41) developed a CADe system for CE video reviewing in real-life clinical setting. For the first time, 2,927 CE videos [OMOM Capsule Endoscopy System (Chongqing Jinshan Science and Technology Co Ltd)], and not only images, from 29 centers were used to train this CNN-based CADe algorithm (SmartScan^®^) to detect and classify 17 different types of small bowel findings. Then, SmartScan was assessed in a validation study comparing conventional reading with CADe-powered reading of 2,898 CE videos from 22 centers (41). SmartScan-assisted reading achieved a significantly higher detection rate of small bowel findings than conventional reading (95.9 vs. 76.1%) in a fraction of the time (mean reading time 5.4 vs. 51.4 min) (41). Altogether, CADe systems are likely to be adopted in CE video analysis increasing detection rate while drastically shortening reviewing time.

## Molecular imaging

Molecular imaging endoscopy combines a high-detailed microscopic visualization with the detecting of specific molecules on the gastrointestinal surface. It requires the topical or intravenous administration of labeled fluorescent agents (42). Once the fluorescent beacon binds the molecular target the two become visible through pCLE (42). This way expression of specific biomarkers in the intestinal mucosa can be assessed *in vivo* or *ex vivo*.

To predict the response to anti-tumor necrosis factor (TNF) treatment, Atreya et al. (43) used fluorescent labeled antibodies against membrane-bound TNF (mTNF) to evaluate *in vivo*, through pCLE, the expression of mTNF in intestinal cells in 25 patients with active CD about to begin therapy with adalimumab. After 12 weeks of treatment, patients with high levels of mTNF-expressing cells had a significantly higher response rate to anti-TNF therapy than those with lower presence of mTNF-expressing cells (92 vs. 15%). Of note, the high mTNF group achieved a sustained clinical remission over a 12-months period with evidence of endoscopic healing at follow-up endoscopy (43).

In a similar fashion, Rath et al. (44) predicted the response to vedolizumab in 5 CD patients with active disease through the topical application of fluorescent labeled antibodies to α4β7 integrin. Then, the mucosal expression of α4β7 integrin was assessed, *ex vivo*, with the use of pCLE. The presence of α4β7+ cells was detected in the two patients who achieved clinical response to Vedolizumab, while no expression of α4β7 integrin was observed in the three non-responders (44).

Despite these promising findings, technical difficulties and impracticality hampers reproducibility and widespread use of this technique. AI might facilitate the analysis of the results and providing a standard interpretation of the intensity of fluorescence signal. Indeed, Iacucci et al. (45) predicted response to biologics in 29 IBD patients using a computerized image analysis of *ex vivo* pCLE with application of fluorescent labeled antibodies directed against TNF and α4β7 integrin.

Molecular imaging could support endoscopists with a real-time visualization of targeted biomarkers, but costs and practical implications have thus far halted its implementation in clinical practice (42).

## AI for histology

As previously mentioned, HR is increasingly considered an important therapeutic target in UC (5). Among UC patients in endoscopic remission, those with histologically active disease are at higher risk of flare and complications (23, 24). Hence, precise and reliable histological analyses are required. Although more than 30 histological scores have been developed, their implementation in real-life clinical practice remains minimal. Even when scores are used, assessment is limited by high interobserver variability. Therefore, operator-independent AI-assisted tools have been proposed as a solution to improve accuracy, standardization and reproducibility.

The first pioneering application of AI to histology in UC came from Vande Casteele et al. (46) who trained a deep learning algorithm to quantify eosinophils in colonic biopsies. This AI model proved a high agreement with manual eosinophil count performed by human pathologists (ICC = 0.81–0.92) in a cohort of 88 UC patients with histologically active disease (46). Although no correlation between AI eosinophil count and histological activity was found, this work paved the way for other studies in the field.

**Figure 1:**
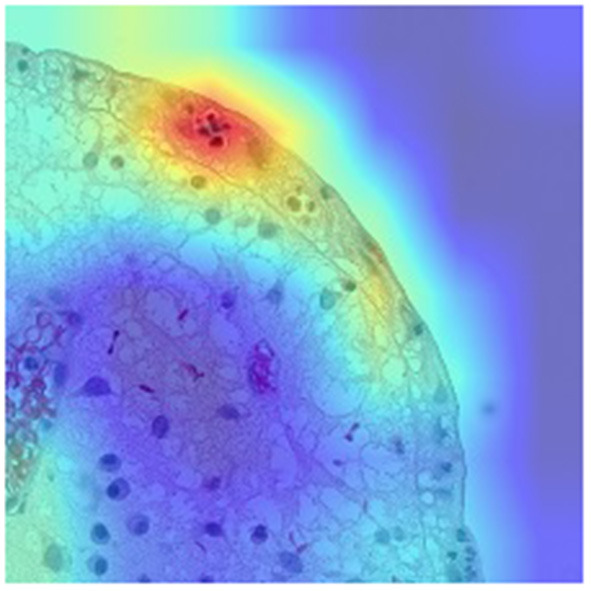
AI-powered assessment of histological activity.

Neutrophils proved to be a better proxy for disease activity than eosinophils. As proposed by Gui et al. (47), neutrophil count alone correlates better than main histological scores to outcomes and endoscopy, while providing higher agreement between pathologists (ICC 0.84). Having overcome the complexity of available histological indices which complicates the development of AI systems, the same group developed an AI model to detect neutrophils and assess remission/activity based on different scores including Nancy, Robarts and PICaSSO Histologic Remission Index (PHRI), which is the newly proposed neutrophil-only score (47) (Figure 1). The CNN-based CAD system, tested on more than 300 biopsies accurately predicted HR (sensitivity 89%, specificity 85% for PHRI and similar for the other scores) (48). Furthermore, the same system stratified the risk of flare similarly to humans, pointing to an evolution of CAD systems providing not only objective assessment but also outcome prediction (48).

Of note, another model developed by Peyrin-Biroulet et al. was recently proposed to assess histological disease activity according to the Nancy index. The CAD was built using a dataset of 200 histological images of UC biopsies (160 for training and 40 for testing) (49) and the preliminary results, presented in 2022 showed that, despite the small sample size, the system and the pathologists had good agreement in assessing the Nancy index (ICC = 0.87). However, crude diagnosis metrics were not reported (49).

These promising results show how operator-independent AI-powered systems could improve, speed up and standardize the histological analysis in clinical practice, leading to better and quicker treatment decisions.

## Discussion

As the treat-to-target strategy has become the “polar star” for IBD management, accurate endoscopic and histological assessment of disease activity is crucial. However, reliable evaluations require prolonged time and specific training, which are not always available. Moreover, even among experienced operators, high interobserver variability significantly affects reliability and reproducibility. In order to address these shortcomings, several studies have focused on the application of AI in IBD endoscopy and histology.

Available evidence showed CAD systems, trained on either videos or images, had a good diagnostic accuracy in evaluating disease activity in UC. AI tools also performed well in predicting HR based on endoscopy. While it is likely that such tools will soon be incorporated in clinical practice, little has been done for CD. Indeed, the patchy and transmural pattern of CD inflammation complicates endoscopic assessment and have so far discouraged studies.

Moreover, AI software could be a great resource for endoscopists for the detection of IBD-associated dysplasia, which remains a challenging task, especially for less experienced operators. Although results in this setting are very preliminary, there are reasons to hope that technology will evolve and we might have CAD systems for detection of dysplasia in IBD patients one day.

Additionally, AI-powered tools have been shown to increase the detection rate of significant findings on CE videos while reducing reviewing time. These progresses may lead to an increase use of CE for proximal small bowel CD assessment, which is more accurate than cross-sectional imaging and better tolerated than double-balloon enteroscopy.

EC and molecular imaging with pCLE are emerging cutting-edge technologies which could potentially revolutionize IBD endoscopy, enormously broadening its capabilities. EC, through an ultramagnified mucosal visualization, could narrow the gap between endoscopy and histology, reducing the need for biopsies. Molecular imaging could also allow a real-time identification of specific targeted biomarkers, which can predict therapeutic response and, thereby, drive therapeutic decisions, paving the way to personalized medicine. Although preliminary studies on EC and molecular imaging have showed promising results, high costs, need for high expertise and poor reproducibility still limit their adoption in daily clinical practice. AI could improve, homogenize and simplify the interpretation of these complex findings, shortening the learning curve.

Finally, beyond the capability of enhancing mucosal evaluation to better predict histological activity, AI models have also been developed for histological assessment, which has gain ground in the last few years. First experiences have shown AI tools could enhance, expedite and standardize histological analysis.

AI, overcoming limitations of human endoscopists, can speed up, standardize and improve endoscopic and histological assessment. Additionally, AI could close the gap between endoscopy and histology, providing a deeper disease characterization without overloading pathologists. Moreover, CAD systems can supervise less experienced operators interpreting complex findings or using advanced technologies, such as EC and molecular imaging, shortening the training curve. Although further validation is eagerly needed, AI is likely to improve IBD assessment and surveillance in the coming future.

Besides all the lights and glitters of AI, some fundamental limitations remain. Computers can assess only what they are presented with, in endoscopy what is scanned. Thus, a careful colonic inspection and an adequate bowel preparation still remain crucial. Despite the rapid progress, CAD systems, just like humans, perform worse with borderline findings, such as very small lesions or mild mucosal inflammation, and thus might not be conclusive.

Furthermore, AI reliability is determined by the dataset these models are trained on. Large and heterogeneous datasets are required to ensure robust training of AI machines, not to incur into overfitting. Which is underperformance of a model when tested in a cohort different from the one it was trained with. Similarly, low prevalence of a condition limits data availability and hence the training of any AI, such as the case of dysplastic lesions in IBD patients.

Another cause of concern related to AI medical applications is the so-called black box problem. In fact, the way an AI output is produced is often inaccessible to the operators; meaning we usually do not know why, what and how the computer processes the information, and in case of a mistake it may be difficult for the users to realize it. Therefore, it would be desirable for clinicians to see how assessment is carried out in order to detect flaws and make the necessary adjustments.

A potential drawback of AI implementation is deskilling, namely the loss of skills due to over-reliance on computers. Similar concerns have been raised for most technological advances and have hardly ever materialized, thus we think AI-caused deskilling does not represent a serious threat to quality of care. Furthermore, the growing role of computer-based decisions will raise legal issues on responsibility. At the moment, the legal framework has not evolved to account for intelligence machines, but in the future, with more autonomous AI devices, manufacturers could be held accountable for errors. Given the foreseeable expansion of “intelligent” tools for diagnosis and treatment, legislators will need to update the legal framework to take into account various degrees of responsibility. Finally, like for any new technology, high costs restrict adoption in resource-limited settings.

In conclusion, numerous exciting technologies promise to revolutionize IBD endoscopy and histology, improving disease characterization and ultimately patient care.

## Author contributions

LA and TP performed the research and wrote the manuscript. RG, AD, AS, CH, MI, AR, and AA critically reviewed the content of the paper. AA conceived the subject of the paper, contributed to the critical interpretation and supervised the project. All authors have read and agreed to the published version of the manuscript.

## Conflict of interest

Author AA received consulting/advisory board fees from AbbVie, Allergan, Amgen, Arena, Biogen, Boehringer Ingelheim, Bristol-Myers Squibb, Celgene, Celltrion, Eli-Lilly, Ferring, Galapagos, Gilead, Janssen, MSD, Mylan, Pfizer, Protagonist Therapeutics, Roche, Samsung Bioepis, Sandoz and Takeda; speaker's fees from AbbVie, Amgen, Arena, Biogen, Bristol-Myers Squibb, Eli-Lilly, Ferring, Galapagos, Gilead, Janssen, MSD, Novartis, Pfizer, Roche, Samsung Bioepis, Sandoz, Takeda and Tigenix and research grants from MSD, Takeda, Pfizer and Biogen. Author AS has served as a speaker, consultant or advisory board member for Ethicon, Takeda, Pfizer, Sofar and Oasis. Authors CH and AR received a consultancy fee from Medtronic and Fujifilm. The remaining authors declare that the research was conducted in the absence of any commercial or financial relationships that could be construed as a potential conflict of interest.

## Publisher's note

All claims expressed in this article are solely those of the authors and do not necessarily represent those of their affiliated organizations, or those of the publisher, the editors and the reviewers. Any product that may be evaluated in this article, or claim that may be made by its manufacturer, is not guaranteed or endorsed by the publisher.
